# Infrared Small Target Detection via Modified Fast Saliency and Weighted Guided Image Filtering

**DOI:** 10.3390/s25144405

**Published:** 2025-07-15

**Authors:** Yi Cui, Tao Lei, Guiting Chen, Yunjing Zhang, Gang Zhang, Xuying Hao

**Affiliations:** 1 Institute of Optics and Electronics, Chinese Academy of Sciences, Chengdu 610209, China; taoleiyan@ioe.ac.cn (T.L.); gtchen2018@163.com (G.C.); yunjingzhang1999@163.com (Y.Z.); zhanggang@ioe.ac.cn (G.Z.); hx_yying@126.com (X.H.); 2 National Laboratory on Adaptive Optics, Chengdu 610209, China

**Keywords:** weighted guided image filter, steering kernel, small target detection, visual saliency

## Abstract

The robust detection of small targets is crucial in infrared (IR) search and tracking applications. Considering that many state-of-the-art (SOTA) methods are still unable to suppress various edges satisfactorily, especially under complex backgrounds, an effective infrared small target detection algorithm inspired by modified fast saliency and the weighted guided image filter (WGIF) is presented in this paper. Initially, the fast saliency map modulated by the steering kernel (SK) is calculated. Then, a set of edge-preserving smoothed images are produced by WGIF using different filter radii and regularization parameters. After that, utilizing the fuzzy sets technique, the background image is predicted reasonably according to the results of the saliency map and smoothed or non-smoothed images. Finally, the differential image is calculated by subtracting the predicted image from the original one, and IR small targets are detected through a simple thresholding. Experimental results on four sequences demonstrate that the proposed method can not only suppress background clutter effectively under strong edge interference but also detect targets accurately with a low false alarm rate.

## 1. Introduction

Infrared small target detection is critical for many military applications, especially for infrared search and tracking systems [1]. Unfortunately, detecting an infrared small target remains a challenging task, primarily due to following reasons: [2] (1) targets are typically small and lack a defined shape or texture, and (2) they are often submerged in a complex background with low signal-to-clutter ratio. Moreover, the background edges exhibiting high contrast and complexity might be falsely detected as targets, which lead to a high false alarm rate [3]. Although numerous methods have been proposed to handle the IR small target detection over the last two decades, it remains an open problem.

Currently, single frame IR small target detection methods can be broadly categorized into four groups: the filter-based model, the human visual system (HVS)-based model, the low-rank and sparse decomposition-based model, and the deep learning-based model. The filter-based approaches, such as the Max-Mean/Max-Median filter [4], the two-dimensional least mean square (TDLMS) filter [5,6], the bilateral filter [7], and the morphological filter [8,9,10,11,12], were developed to suppress background and clutter. They are mostly simple and fast but always enhance the edges of the background and make it difficult to determine whether it is the target or the edge during the detection process [2]. Leveraging the low-rank property of the background and the sparsity of the targets, the infrared patch image (IPI) model [1] pioneered the low-rank and sparse decomposition-based methods. Subsequently, numerous efforts have focused on improving its performance, such as the weighted infrared patch image (WIPI) model [13], the non-convex rank approximation minimization joint l_2,1_ norm (NRAM) [14], the partial sum of the tensor nuclear norm (PSTNN) [15], target-aware non-local low-rank modeling with saliency filtering regularization (TNLRS) [2], the image patch tensor model (IPT) [16], nonconvex tensor fibered rank approximation (NTFRA) [17], and the edge and corner awareness-based spatial-temporal tensor (ECA-STT) [18] model. Although the previously mentioned methods WIPI [13] and NRAM [14] have made significant progress to remove the edge residuals, they cannot fully eliminate the strong local clutters of various shapes completely by employing only a specific sophisticated norm to replace the nuclear norm [19]. Separately, HVS-based methods have been proposed regarding the dissimilarity between the current location and its neighborhood; these include the local contrast measure (LCM) [20], multi-scale patch based contrast measure (MPCM) [21], relative local contrast measure (RLCM) [22], derivative entropy-based contrast measure (DECM) [23], tri-layer local contrast measure (TLLCM) [24], double-neighborhood gradient method (DNGM) [25], strengthened robust local contrast measure (SRLCM) [26], and adaptive scale patch-based contrast measure (ASPCM) [27]. The above models usually adopt a sliding window to traverse the original image and have a fast-running speed, but most of them fail to maintain high performance when encountering a heterogeneous background exhibiting similar characteristics across local regions [2,28]. Recently, due to its automatic feature-learning ability for image hierarchical features, deep learning has been widely used for small target detection, such as adversarial learning for small object segmentation [29], the robust infrared small target detection network (RISTDnet) [30], the infrared small target detection with generative adversarial network (IRSTD-GAN) [31], the attentional local contrast network (ALCNet) [32], and the cross-connected bidirectional pyramid network (CBP-Net) [33]. However, the learning-based approaches are not universally applicable in IR small target detection, perhaps due to factors such as limited training data or requirements for predictability in novel scenarios.

Specifically, the spatial domain filter-based technique, which seeks to predict the background as accurately as possible, is a simple yet effective tool for infrared small target detection. Within this technique, the background estimation is the most important step, usually conducted as follows: [34] filtering is first applied locally centered on each pixel. The original pixel value is then replaced with the calculated result; this process is repeated for the entire image to generate the background prediction image. An essential consideration for background estimation is the need to preserve various edges in the background, since edges are highly prone to producing false alarms. However, most of the current spatial filter-based methods perform well on smooth backgrounds but yield less satisfactory results when encountering complex scenes containing different kinds of edges, such as the above-mentioned Max-Mean/Max-Median filter [4], Top-Hat filter [8], and TDLMS filter [5]. To overcome this shortcoming, Bae et al. [6,7] proposed edge directional bilateral/TDLMS filter-based methods, utilizing the edge information of surrounding prediction pixels in four directions. These methods can work well when the edges are obvious and strong, while it is still inadequate to cope with sophisticated edges, which are complex and vary in the real scenes.

As mentioned above, a crucial challenge in small target detection is to recover objects as much as possible while distinguishing them from the most representative clutters of the image, such as edges, corners, and other sharp structures. To solve this problem, we proposed a background estimation method for small target detection via fast saliency [35] and WGIF [36]. This method can eliminate or reduce edges of varying degrees and directions effectively and extract real targets under complex natural scenes. In addition, the proposed algorithm is suitable for parallel processing, which is useful for improving the detection speed. To the best of our knowledge, the proposed approach is the first work that combines the advantage of WGIF and fast visual saliency for infrared small target detection. Notably Ref. [37] applied the guided image filter to IR small target detection, but only employed it as a preprocessing step to strengthen the low-rankness of background components and suppress noise components.

The rest of this paper is organized as follows. The second section describes the proposed method in detail, followed by experimental results and analysis in the third section. We demonstrate the performance of the proposed method by comparing it with other SOTA methods. Finally, the paper is concluded in the fourth section.

## 2. Proposed Algorithm

**Figure 1:**
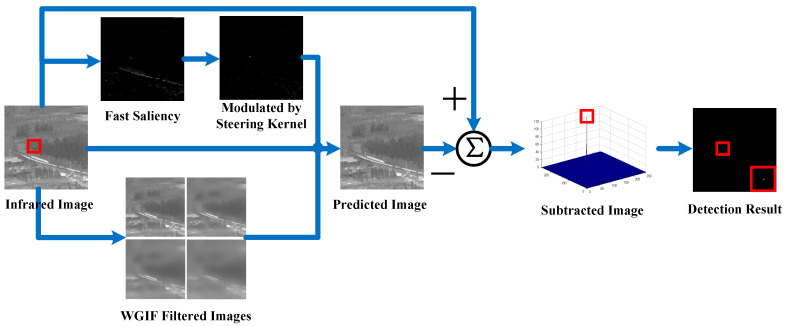
Illustration of the proposed method (targets are shown in red rectangles).

Figure 1 illustrates the complete flowchart of the proposed infrared small target detection method. The fast saliency map is derived using the facet kernel described in Ref. [35]. Subsequently, the steering kernel [38] is utilized to remove residual strong edges. The modulated saliency map represents the degree of the smoothing effect for each pixel. On the other hand, the edge-preserving smoothed images are acquired from a series of WGIF operations. Then the predicted background image pixel value is computed via fuzzy sets from the smoothed or non-smoothed images according to the previous modulated saliency map. Finally, the target is extracted from the subtracted image by applying simple thresholding.

### 2.1. Saliency Map Calculation

Since the conventional spatial domain filter is not designed specifically for small target detection, a saliency map representing the probability of small target presence is delicately constructed for the subsequent background prediction process. This enables more accurate region classification.

Firstly, the gradient magnitude map *R* is obtained by convolving the original infrared image *D* with the 5 × 5 facet kernel *F* [35] as follows:

(1) $$R=D\times F,F=\left(\begin{array}{ccccc} -4 & -1 & 0 & -1 & -4\\ -1 & 2 & 3 & 2 & -1\\ 0 & 3 & 4 & 3 & 0\\ -1 & 2 & 3 & 2 & -1\\ -4 & -1 & 0 & -1 & -4 \end{array}\right)$$

The square value for each pixel is computed in the map *R* to get the enhanced map *E*:

(2) $$E=R^{2}$$

Typically the most salient point in the enhanced map *E* is a target. However, it still contains numerous edge interferences as shown in the fast saliency map of Figure 1. Thus, this result should be refined to increase confidence in the small target presence.

The steering kernel is an attractive tool in image processing [38]. Extensive experiments have shown that feature descriptors using SK are robust to brightness variation and noise interference. Unfortunately, the SK descriptor of a small-target patch highly resembles that of a texture clutter patch but differs dramatically from a structural edge region [39]. Hence, we use SK to suppress residual edges in the previous enhanced map *E*.

The descriptive power of the SK mainly derives from a symmetric gradient covariance matrix $C_{i}$, which can be estimated as:

(3) $${\hat{C}}_{i}\approx G_{i}^{T}G_{i},G_{i}=\left[\begin{array}{cc} \vdots & \vdots \\ D_{k,x_{1}} & D_{k,x_{2}}\\ \vdots & \vdots \end{array}\right],\forall k\in \omega_{i}$$

Here, $G_{i}$ is the local gradient matrix, $\omega_{i}$ is the square window centered at pixel *i*, and $D_{k,x_{1}}$ and $D_{k,x_{2}}$ are the first derivatives along $x_{1}$ and $x_{2}$ directions at pixel *k*, which are computed by the second order classic kernel regression method. To improve robustness and stability, the gradient covariance matrix is decomposed into three components as follows [38]:

(4) $$C_{i}=\gamma_{i}U_{\theta_{i}}\Lambda_{i}U_{\theta_{i}}^{T}$$

(5) $$U_{\theta_{i}}=\left[\begin{array}{cc} \cos\theta_{i} & \sin\theta_{i}\\ -\sin\theta_{i} & \cos\theta_{i} \end{array}\right]$$

(6) $$\Lambda_{i}=\left[\begin{array}{cc} \sigma_{i} & 0\\ 0 & \sigma_{i}^{-1} \end{array}\right]$$

where $U_{\theta_{i}}$ is a rotation matrix and $\Lambda_{i}$ is the elongation matrix. The elongation parameter $\sigma_{i}$, scaling parameter $\gamma_{i}$, and rotation parameter $\theta_{i}$ are all determined by the singular value decomposition (SVD) of the local gradient matrix $G_{i}$. If the diagonal matrix of SVD is denoted as $diag(s_{1},s_{2})$, the elongation parameter $\sigma_{i}$ corresponding to the energy of the dominant gradient direction is denoted as:

(7) $$\sigma_{i}=\frac{s_{1}+\lambda^{\prime }}{s_{2}+\lambda^{\prime }},\lambda^{\prime }\geq 0$$

The scaling parameter $\gamma_{i}$ is given as:

(8) $$\gamma_{i}={\left(\frac{s_{1}s_{2}+\lambda \prime \prime }{M}\right)}^{\frac{1}{2}}$$

where $\lambda^{\prime }$ and λ″ are regularization parameters, and M is the size of local patch. Considering the meanings of the elongation and scaling parameters, similar to Ref. [13], the edge unlikelihood coefficient is constructed as follows:

(9) $$p_{i}=\frac{1}{\sigma_{i}\gamma_{i}}$$

Consequently, the SK modulated saliency map *S* is defined as:

(10) S=E⊙P

Leveraging the structurally informative edge unlikelihood coefficient, the proposed SK modified saliency map *S* effectively eliminates prominent edges while preserving targets, as shown in Figure 1.

### 2.2. Weighted Self-Guided Image Filtering

By incorporating an edge-aware weighting into an existing guided image filter [40], the WGIF [36] is introduced to produce images with excellent visual quality and avoid halo artifacts like the existing global smoothing filters. Due to the WGIF’s outstanding edge-preserving smoothing property, we adopted this method to estimate the candidate background for small targets. Since the guided image is the same as the input infrared image *D*, we refer to this self-guided image filtering.

Let $Var_{k}$ be the variance of *D* in the 11 × 11 local window; an edge-aware weighting $w_{k}$ is defined for all pixels as follows:

(11) $$w_{k}=\frac{1}{N}\sum_{k^{\prime }=1}^{N}\frac{Var_{k}+\varepsilon }{Var_{k^{\prime }}+\varepsilon }$$

where *N* is the number of pixels; ε is a small constant, and its value is selected as ${\left(0.001\times L\right)}^{2}$; while *L* is the dynamic range of the image *D*. It should be pointed out that, taking into account the common scale of the small target, the size of the local window is somewhat larger than the value recommended in Ref. [36].

The linear coefficients $\left(a_{k},b_{k}\right)$ centered at pixel *k* assumed to be constant in a square window of a radius *r* (denoted as $\omega_{k}$) are computed as follows:

(12) ak=δk2δk2+λwkbk=(1−ak)μk

where $\mu_{k}$ and $\delta_{k}^{2}$ are the mean and variance of *D* in $\omega_{k}$, λ is a regularization parameter, and $w_{k}$ is the weighting mentioned in Equation (11).

After computing $\left(a_{k},b_{k}\right)$ for all windows $\omega_{k}$ in the image, the filtering output $q_{i}$ centered at pixel *i* is given as:

(13) $$\begin{array}{l} {\bar{a}}_{i}=\frac{1}{\left|\omega \right|}\sum_{k\in \omega_{i}}a_{k}\\ {\bar{b}}_{i}=\frac{1}{\left|\omega \right|}\sum_{k\in \omega_{i}}b_{k}\\ q_{i}={\bar{a}}_{i}D_{i}+{\bar{b}}_{i} \end{array}$$

Here, ${\bar{a}}_{i}$ and ${\bar{b}}_{i}$ are the average coefficients of all windows overlapping i, and $\left|\omega \right|$ is the number of pixels in $\omega_{i}$.

The WGIF is applied repeatedly for the input image D with four different sets of parameters $\left(r_{j},\lambda_{j}\right)\left(j=1,2,3,4\right)$, and the edge-preserving images with vary smoothing effect are acquired easily, denoted as $G_{j}\left(j=1,2,3,4\right)$, as shown in Figure 1.

### 2.3. Background Prediction Using Fuzzy Sets

To enhance WGIF’s performance specifically in small target detection, we can utilize prior knowledge from the saliency map *S* to facilitate the background estimation process. Note that the value of the saliency map *S* represents the probability of a target; the mapping relationship between the target prior distribution and the WGIF filtered/non-filtered images could be established to generate a background image *B*. Partially inspired from Ref. [7], the background reconstruction mechanism is heuristically defined by the following rules:

If a pixel’s saliency is low, then the predicted pixel is chosen from less smoothed filtered image.

If a pixel’s saliency is medium, then the predicted pixel is chosen from medium smoothed filtered image.

If a pixel’s saliency is high, then the predicted pixel is chosen from more smoothed filtered image.

**Figure 2:**
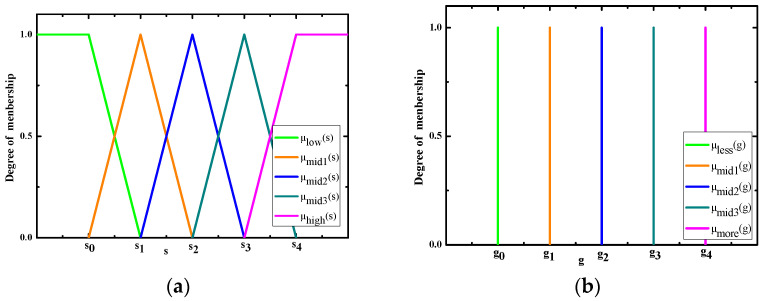
Input (**a**) and output (**b**) membership functions for background prediction.

Since these are fuzzy terms, we can express the concepts of saliency and smoothing extent by the membership functions. As shown in Figure 2, the triangular and singleton types of membership functions are defined for input saliency and output pixel gray, respectively. A serial of parameters $s_{j}(j=0,1,2,3,4)$ and $g_{j}(j=0,1,2,3,4)$ are applied for the construction of the membership functions. It should be noted that the value of $g_{0}$ is set the same as the original image pixel gray, and others $g_{1},g_{2},g_{3},g_{4}$ come from WGIF filtered images $G_{j}\left(j=1,2,3,4\right)$ obtained by different smooth parameters $\left(r_{j},\lambda_{j}\right)\left(j=1,2,3,4\right)$. Considering the processing speed is an important factor for small target detection, the constant output membership function significantly reduces computational requirements. Because we are dealing with constants in the output membership function, the output predicted background image, $B_{i}$, to any saliency map input, $s_{i}$, is given by:

(14) $$B_{i}=\frac{\mu_{low}\left(s_{i}\right)\times g_{0}+\mu_{mid1}\left(s_{i}\right)\times g_{1}+\mu_{mid2}\left(s_{i}\right)\times g_{2}+\mu_{mid3}\left(s_{i}\right)\times g_{3}+\mu_{high}\left(s_{i}\right)\times g_{4}}{\mu_{low}\left(s_{i}\right)+\mu_{mid1}\left(s_{i}\right)+\mu_{mid2}\left(s_{i}\right)+\mu_{mid3}\left(s_{i}\right)+\mu_{high}\left(s_{i}\right)}$$

According to Equation (14), the background image *B* is constructed pixel by pixel from the corresponding smoothed or non-smoothed images. When the pixel lies in a target region with a high saliency value, the background pixel is computed from the corresponding WGIF filtered images with both high radius r and regularization parameter λ, thus giving a more intensive blurring effect to the potential target region. Conversely, when the pixel lies in flat or edge locations, the predicted pixel is obtained from the less smoothed images or retained directly from the original image. Leveraging WGIF’s excellent edge-preserving smoothing performance, the major image structures and the complex edge clutter are retained in the background component, while the small target is clearly removed. The predicted image is displayed in Figure 1.

### 2.4. Target Detection

A subtraction operation is adopted to separate the target from the complex background:

(15) T=D−B

In the subtracted image *T*, the target becomes evident while background clutter is significantly suppressed. Subsequently, the binarization is carried out by setting the threshold as $\alpha T_{\max}$, where $T_{\max}$ is the maximum grayscale of the subtracted image and α is an adjustment parameter of the threshold. Our experiments show that setting α as 0.3~0.5 is sufficiently high for single-target segmentation since the target is very salient and the clutter is well suppressed.

## 3. Experimental and Analysis

In this section, we validate the effectiveness of the proposed approach using four real IR image sequences. Seq. 1 belongs to the cloudy sky scenario, and Seq.2~Seq.4 [41] are IR image data sets with complex backgrounds. The descriptions of each sequence used for performance evaluation are listed in Table 1. All the experiments were implemented in MATLABR2014a and run on a computer with 8 GB memory and IntelCorei7-8565U CPU. The filter radius and regularization parameters $\left(r_{j},\lambda_{j}\right)\left(j=1,2,3,4\right)$ in WGIF procedure were set as $\left(4,0.1^{2}\right),\left(8,0.2^{2}\right),\left(12,0.4^{2}\right),\left(16,0.8^{2}\right)$, respectively. The fuzzy set parameters $s_{j}\left(j=0,1,2,3,4\right)$ related to background prediction were set empirically as 0.4, 0.5, 0.6, 0.7, 0.8. It is worth noting that the choice of parameters in the above discussion appears to be purely ad hoc; we could also adjust these values carefully to achieve a better detection result.

### 3.1. Evaluation Metrics and Comparison Methods

To evaluate the performance of the proposed method, the signal-to-clutter ratio gain (SCRG), the background suppression factor (BSF), and the contrast gain (CG) are introduced as:

(16) $$\mathrm{SCRG}=\frac{{\mathrm{SCR}}_{\mathrm{out}}}{{\mathrm{SCR}}_{\mathrm{in}}},\mathrm{BSF}=\frac{C_{\mathrm{in}}}{C_{\mathrm{out}}},\mathrm{CG}=\frac{{\mathrm{CON}}_{\mathrm{out}}}{{\mathrm{CON}}_{\mathrm{in}}}$$

where ${\mathrm{SCR}}_{\mathrm{in}}$, $C_{\mathrm{in}}$, and ${\mathrm{CON}}_{\mathrm{in}}$ are the signal-to-clutter ratio (SCR), the standard deviation of the entire background, and the contrast measure of the input image, respectively;${\mathrm{SCR}}_{\mathrm{out}}$, $C_{\mathrm{out}}$, and ${\mathrm{CON}}_{\mathrm{out}}$ denote the corresponding values for the processed image. The definitions of SCR and CON are given as:

(17) $$\mathrm{SCR}=\frac{\left|\mu_{t}-\mu_{b}\right|}{\sigma_{b}},\mathrm{CON}=\left|\mu_{t}-\mu_{b}\right|$$

where $\mu_{t}$, $\mu_{b}$, and $\sigma_{b}$ are the gray average of the target, the gray average of the background, and the standard deviation of the local background, respectively. It is noted that the sequence averages of the above-mentioned metrics calculated in this paper are denoted as $\bar{\mathrm{SCRG}}$, $\bar{\mathrm{BSF}}$, and $\bar{\mathrm{CG}}$. The other two important metrics are the detection probability $P_{d}$ and the false alarm rate $F_{a}$, which are defined as:

(18) $$P_{d}=\frac{\mathrm{number}\mathrm{of}\mathrm{true}\mathrm{detections}}{\mathrm{number}\mathrm{of}\mathrm{actural}\mathrm{targets}}$$

(19) $$F_{a}=\frac{\mathrm{number}\mathrm{of}\mathrm{false}\mathrm{detections}}{\mathrm{number}\mathrm{of}\mathrm{images}}$$

In this paper, the local contrast measure (LCM) [20], multiscale patch-based contrast measure (MPCM) [21], tri-layer local contrast measure (TLLCM) [24], local intensity and gradient (LIG) [42], infrared patch image (IPI) [1], partial sum of the tensor nuclear norm (PSTNN) [15], and nonconvex tensor fibered rank approximation (NTFRA) [17] are chosen for comparisons. The parameter settings of all compared methods are listed in Table 2.

### 3.2. Experimental Results and Analysis

**Figure 3:**
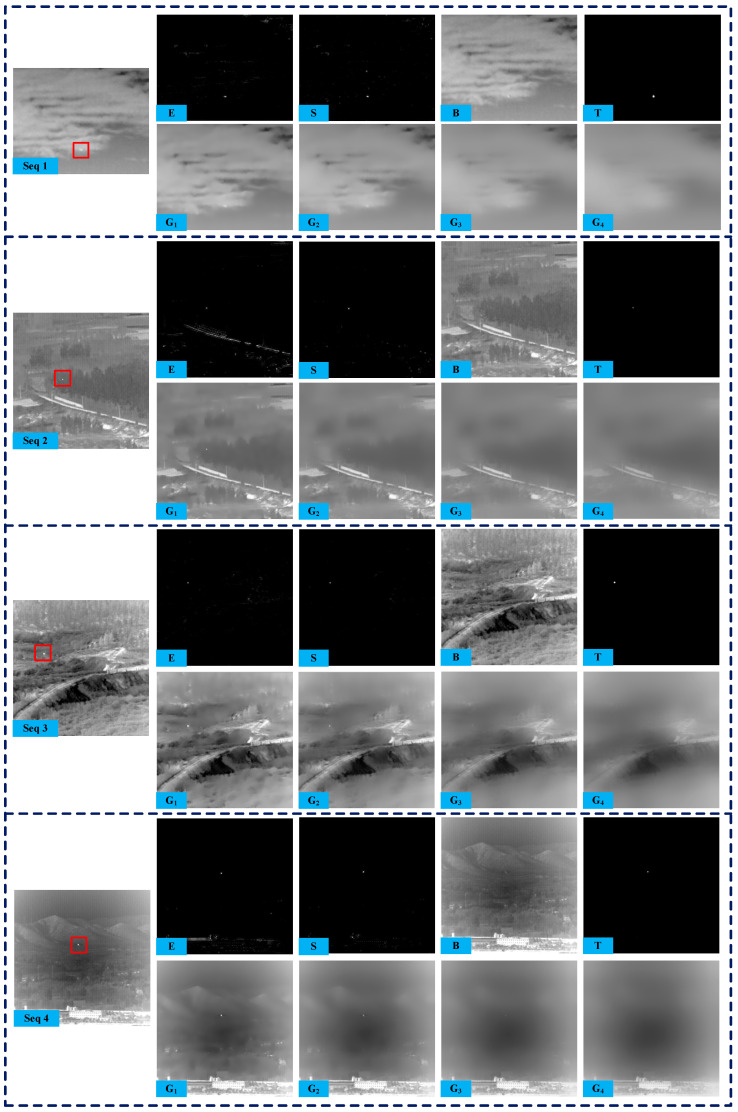
The results of the fast saliency map E, the SK modulated saliency map S, the WGIF smoothed images Gj, the predicted image B, and the subtracted image T on four real IR sequences (red rectangle marks a true target in the original images).

To manifest the process clearly, the detailed intermediate results obtained by the proposed method are shown in Figure 3. These include the fast saliency map *E*, the SK modulated saliency map *S*, the WGIF smoothed images $G_{j}\left(j=1,2,3,4\right)$, the predicted image *B*, and the subtracted image *T*. By comparing *E* and *S* in the same sequence, we can see most of the strong edge responses produced by fast saliency are removed correctly through the SK module. In addition, the different WGIF filtering results demonstrate both the edge-preserving effect and the smoothing of small targets. It is also clearly evident that the small target is nearly eliminated from the reconstructed background image *B*. Therefore, the small target can be easily detected through the simple subtraction operation.

To evaluate the contribution of the critical components to the proposed method, an ablation experiment is conducted to illustrate the effectiveness of the steering kernel and edge-aware weighting. Table 3 shows that the results obtained by non-SK modified fast saliency and non-weighted guided image filtering are inferior to those of the full-components method for all the sequences evaluated. Therefore, the combination of SK and WGIF significantly reduces complex edges and enhances target detection while suppressing backgrounds.

There are several key parameters such as the filter radii, regularization parameters, and fuzzy set parameters. To achieve better performance with real datasets, it is necessary to adjust these parameters carefully. Six groups of parameters and the chosen set are listed in Table 4. The average SCRG, BSF, and CG corresponding to different parameters are shown in Table 5 for Sequences 1–4. It should be noted that each parameter was tuned while keeping the others fixed, potentially resulting in suboptimal performance. From the results of Groups 1–4, it is observed that the algorithm was relatively insensitive to the variations in filter parameters, as different filter radii and regularization parameters all performed well on the four sequences. Furthermore, Group 5 achieved the best CG values for all the sequences but yielded the worst results for SCRG and BSF. This phenomenon may be attributed to the larger step size between the fuzzy parameters, which enhances object contrast. Therefore, to achieve an overall balanced performance, we adopt the moderate step size of filter parameters and fuzzy set parameters, as shown in Table 4.

**Figure 4:**
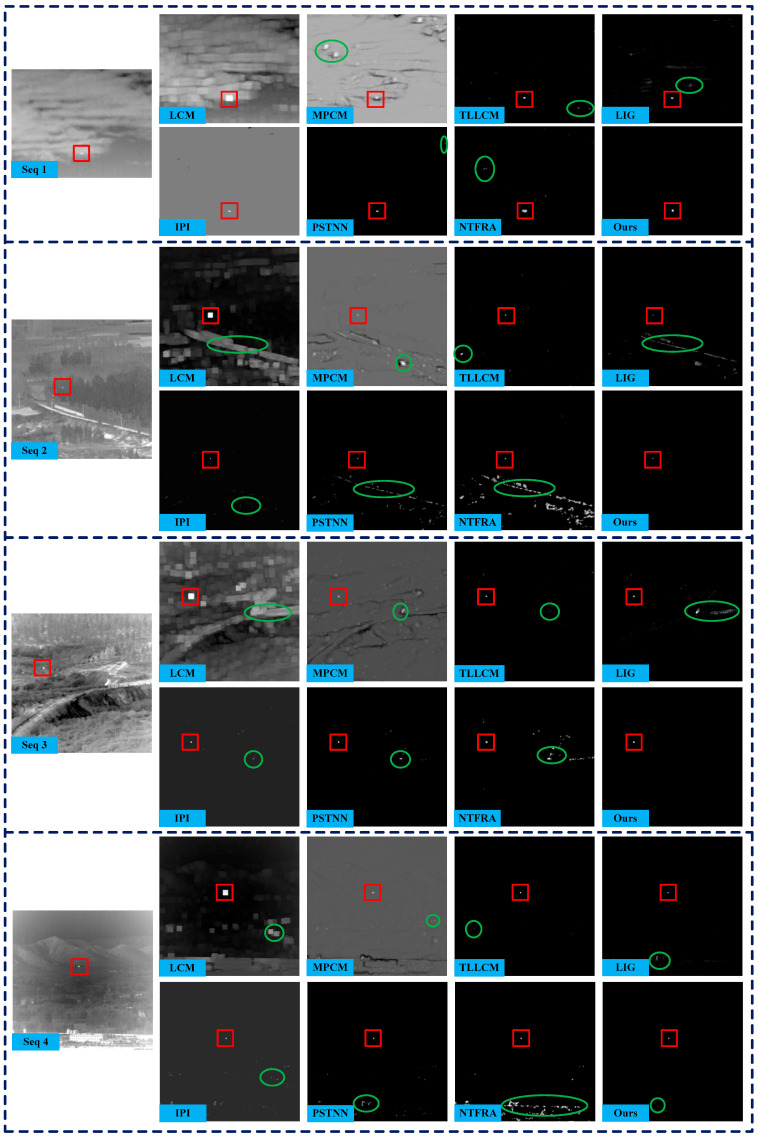
Original images and experimental results of different methods on four real IR sequences (red rectangle marks a true target, and green ellipse denotes a false alarm location).

**Figure 5:**
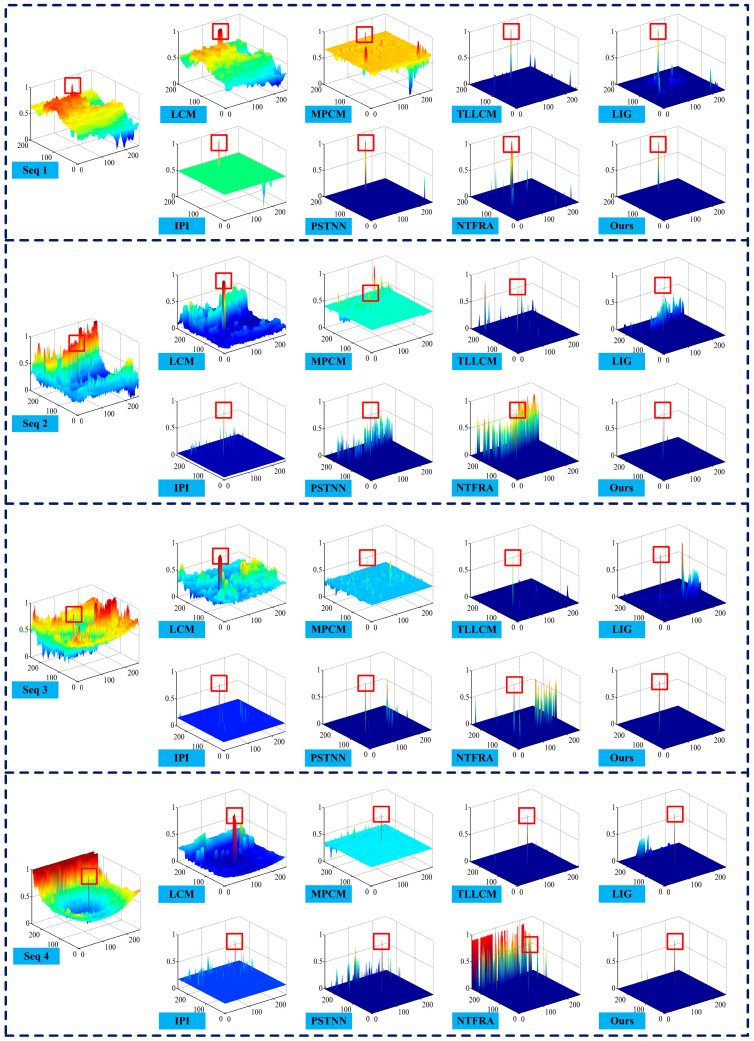
Comparison of 3D gray distributions by different methods on four real IR sequences (red rectangle marks a true target).

For visual comparison, representative images from the four sequences and detection results, including the corresponding 3D gray distributions obtained by different algorithms, are given in Figure 4 and Figure 5. The results of LCM, MPCM, and NTFRA contain the most residual background, indicating that their background suppression capability is inferior to the other methods. LIG and PSTNN show some progress in background suppression, but both exhibit significant responses to strong edges, which is particularly evident in Seq. 2. Although IPI and TLLCM effectively suppress the background and detect the targets, the false alarms nevertheless occur in Seqs. 2, 3, and 4. In contrast, our method extracts the targets correctly and removes almost all background on the four sequences except some faint residual clutter in Seq. 4. These experimental results visually demonstrate that the proposed method has excellent background suppression capability, especially against edge clutters under complex scenes.

The average SCRG, BSF, CG, and processing time of each method are listed in Table 6. The assessment criteria SCRG and BSF evaluate the performance of local background suppression and target enhancement. The CG is adopted because in some cases the other two metrics may be very large or infinite [43]. Higher values for all three criteria indicate better performance. To be more intuitive, the best result is marked in bold. As shown, the proposed method achieves the best SCRG and BSF for all four sequences, indicating superior background suppression performance. Although no contrast stretching transformation is applied to enhance the subtracted image, our method still maintains the largest CG values in Seq. 3 and Seq. 4, suggesting better target enhancement capability. In terms of processing time, the proposed method is not as efficient as the LCM or MPCM, but it possesses significant potential for parallel processing and could be readily accelerated using the multi-thread technique or even GPU implementation.

**Figure 6:**
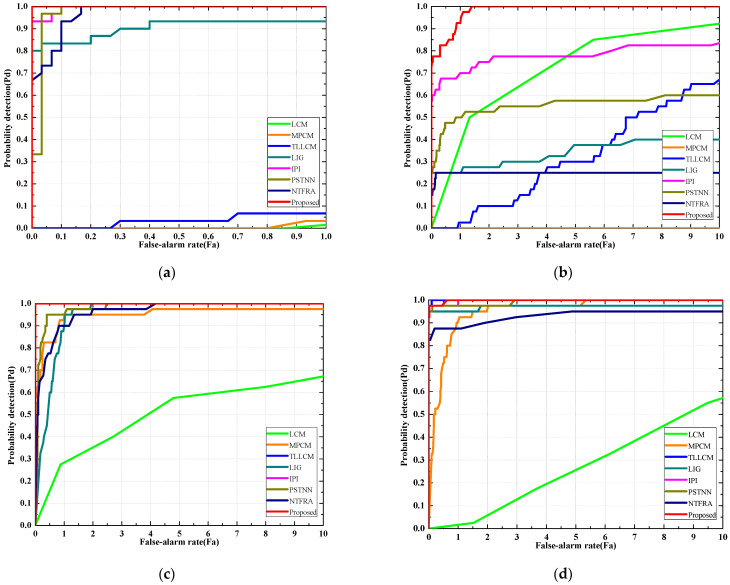
ROC curves of four sequences. (**a**) Seq. 1. (**b**) Seq. 2. (**c**) Seq. 3. (**d**) Seq. 4.

To demonstrate the robustness of our method comprehensively, the receiver operating characteristic (ROC) curves of different methods are shown in Figure 6. These curves illustrate the tradeoff between the detection probability and the false alarm rate. The results indicate that our method is advantageous over other methods in most cases and achieves the best overall detection performance among the eight methods. However, it is noteworthy that TLLCM achieves an impressive detection performance in Seq. 4. The main reason lies in the more evident contrast between the target and surrounding background in Seq. 4 compared to the other sequences, combined with the TLLCM’s ability to first enhance the target, thereby making the distinction sufficiently obvious. This explains why TLLCM’s performance is better than that of LCM or MPCM in most cases. Additionally, the ROC curves of TLLCM, IPI, and the proposed method are nearly overlapping in Seq. 3, indicating comparable performance among these three methods.

## 4. Conclusions

This paper proposes an IR small target detection method based on modified fast saliency and WGIF to achieve enhanced detection performance under cluttered background. First, by calculating the SK modified saliency map, a simple yet powerful image prior is introduced to characterize targets, modeled as the target existence probability. Then, the edge-preserving smoothed images are acquired by utilizing the rapid local spatial filter WGIF. Subsequently, the background is estimated based on a mapping relationship between the saliency map and smoothed/original images, constructed via the fuzzy set technique. Finally, the target is easily extracted by subtraction and thresholding. The experimental results demonstrate that the proposed method not only achieves a good detection performance and edge suppression ability but also outperforms other baseline methods in various scenarios.

## Figures and Tables

**Table 1:** Description of four sequences.

Sequence	Size/Pixels	Length/Frames	Target Description	Background Description
Seq.1	256 × 200	30	Single, relatively large, moving along cloud edges	Heavy cloud sky
Seq.2	256 × 256	80	Single, tiny, low nonlocal contrast	Complex road and forest
Seq.3	256 × 256	80	Single, tiny, varying size	Much target-like clutter
Seq.4	256 × 256	80	Single, a little long strip, varying size	Mountain and artificial structures

**Table 2:** Parameters of 8 methods.

No.	Method	Parameter Settings
1	LCM	Largest scale *S =* 4 size: 3 × 3, 5 × 5, 7 × 7, 9 × 9
2	MPCM	Mean filter size: 3 × 3, *N* = 3,5,7,9
3	TLLCM	Core layer size: 3 × 3, Reserve layer size: 5 × 5, 7 × 7, 9 × 9
4	LIG	Sliding window size: 11 × 11, *k* = 0.2
5	IPI	Patch size: 50 × 50, step:10 $\lambda =1/\sqrt{\min(n_{1},n_{2})},\varepsilon =10^{-7}$
6	PSTNN	Patch size: 40 × 40, step:40 $\lambda =0.7/\sqrt{\min(n_{1},n_{2})*n_{3}},\varepsilon =10^{-7}$
7	NTFRA	Patch size: 40 × 40, step:40 $\lambda =1/\sqrt{\min(n_{1},n_{2})*n_{3}},\beta =0.01,\mu =200$
8	Proposed	Fuzzy set parameters: 0.4,0.5,0.6,0.7,0.8 $\mathrm{Filter parameters:}\left(4,0.1^{2}\right),\left(8,0.2^{2}\right),\left(12,0.4^{2}\right),\left(16,0.8^{2}\right)$

**Table 3:** The impact of different components on the performance of the proposed method.

	Methods	Without SK	Without Weighting	Proposed
$\bar{\mathrm{SCRG}}$	Seq 1	Inf	Inf	Inf
	Seq 2	Inf	Inf	Inf
	Seq 3	Inf	Inf	Inf
	Seq 4	Inf	Inf	Inf
$\bar{\mathrm{BSF}}$	Seq 1	Inf	Inf	Inf
	Seq 2	9.339	24.959	Inf
	Seq 3	Inf	Inf	Inf
	Seq 4	36.312	48.393	58.503
$\bar{\mathrm{CG}}$	Seq 1	2.368	2.728	3.198
	Seq 2	0.768	1.455	1.510
	Seq 3	0.904	0.830	1.767
	Seq 4	0.991	1.163	1.488

**Table 4:** Six groups of experimental parameters and the finally adopted ones.

No.	Filter Parameters	Fuzzy Set Parameters
Group 1	$\left(4,0.1^{2}\right),\left(6,0.2^{2}\right),\left(8,0.4^{2}\right),\left(10,0.8^{2}\right)$	0.4,0.5,0.6,0.7,0.8
Group 2	$\left(4,0.1^{2}\right),\left(10,0.2^{2}\right),\left(16,0.4^{2}\right),\left(22,0.8^{2}\right)$	0.4,0.5,0.6,0.7,0.8
Group 3	$\left(4,0.1^{2}\right),\left(8,0.15^{2}\right),\left(12,0.2^{2}\right),\left(16,0.25^{2}\right)$	0.4,0.5,0.6,0.7,0.8
Group 4	$\left(4,0.1^{2}\right),\left(8,0.5^{2}\right),\left(12,1.0^{2}\right),\left(16,1.5^{2}\right)$	0.4,0.5,0.6,0.7,0.8
Group 5	$\left(4,0.1^{2}\right),\left(8,0.2^{2}\right),\left(12,0.4^{2}\right),\left(16,0.8^{2}\right)$	0.1,0.3,0.5,0.7,0.9
Group 6	$\left(4,0.1^{2}\right),\left(8,0.2^{2}\right),\left(12,0.4^{2}\right),\left(16,0.8^{2}\right)$	0.5,0.55,0.6,0.65,0.7
Adopted	$\left(4,0.1^{2}\right),\left(8,0.2^{2}\right),\left(12,0.4^{2}\right),\left(16,0.8^{2}\right)$	0.4,0.5,0.6,0.7,0.8

**Table 5:** Average SCRG, BSF, and CG by different parameters of the proposed method.

	Parameters	Group 1	Group 2	Group 3	Group 4	Group 5	Group 6	Adopted
$\bar{\mathrm{SCRG}}$	Seq 1	Inf	Inf	Inf	Inf	Inf	Inf	Inf
	Seq 2	Inf	Inf	Inf	Inf	173.22	Inf	Inf
	Seq 3	Inf	Inf	Inf	Inf	Inf	Inf	Inf
	Seq 4	Inf	Inf	Inf	Inf	106.05	Inf	Inf
$\bar{\mathrm{BSF}}$	Seq 1	Inf	Inf	Inf	Inf	Inf	Inf	Inf
	Seq 2	114.73	90.617	129.08	67.432	21.977	Inf	Inf
	Seq 3	Inf	Inf	Inf	Inf	107.01	Inf	Inf
	Seq 4	162.51	49.14	45.647	380.51	38.99	Inf	58.503
$\bar{\mathrm{CG}}$	Seq 1	3.167	3.235	3.006	2.967	3.346	3.099	3.198
	Seq 2	1.469	1.493	1.486	1.518	1.681	1.418	1.510
	Seq 3	1.691	1.739	1.717	1.787	1.867	1.662	1.767
	Seq 4	1.311	1.451	1.541	1.468	1.566	1.407	1.488

**Table 6:** Average SCRG, BSF, CG, and processing time obtained by 8 methods on four sequences.

	Methods	LCM	MPCM	TLLCM	LIG	IPI	PSTNN	NTFRA	Proposed
$\bar{\mathrm{SCRG}}$	Seq 1	1.563	1.656	Inf	52.325	9.130	Inf	8.875	Inf
	Seq 2	1.638	1.496	17.972	131.880	Inf	Inf	Inf	Inf
	Seq 3	0.533	2.131	Inf	23.649	7.656	Inf	4.330	Inf
	Seq 4	0.320	1.695	4.394	38.384	5.106	Inf	6.140	Inf
$\bar{\mathrm{BSF}}$	Seq 1	0.706	2.135	13.132	10.070	16.186	Inf	10.875	Inf
	Seq 2	0.889	4.097	8.151	3.895	14.771	3.323	1.309	Inf
	Seq 3	1.612	6.904	29.250	10.141	34.615	15.666	4.999	Inf
	Seq 4	1.984	7.818	42.535	12.850	19.539	10.391	2.645	58.503
$\bar{\mathrm{CG}}$	Seq 1	3.306	1.172	2.264	2.857	2.591	3.328	4.320	3.198
	Seq 2	3.972	0.888	1.837	1.219	1.235	1.482	1.911	1.510
	Seq 3	1.421	0.661	1.309	1.464	1.261	1.575	1.729	1.767
	Seq 4	1.475	0.775	1.332	1.306	1.161	1.408	1.407	1.488
$\bar{\mathrm{Time}}/s$	Seq 1	0.0786	0.0837	2.041	1.270	6.311	0.0634	1.211	0.715
	Seq 2	0.0902	0.0901	2.773	1.647	8.553	0.282	1.827	0.894
	Seq 3	0.0877	0.0899	2.864	1.667	9.007	0.229	1.902	0.968
	Seq 4	0.0899	0.0925	2.752	1.674	10.055	0.253	1.793	0.901

## Data Availability

The original contributions presented in this study are included in the article. Further inquiries can be directed to the corresponding author(s).
